# Geography, taxonomy, and ecological guild: Factors impacting freshwater macroinvertebrate gut microbiomes

**DOI:** 10.1002/ece3.9663

**Published:** 2022-12-23

**Authors:** Paul A. Ayayee, Jeff S. Wesner, Scot P. Ouellette

**Affiliations:** ^1^ Department of Biology University of Nebraska at Omaha Omaha Nebraska USA; ^2^ Department of Biology University of South Dakota Vermillion South Dakota USA; ^3^ Department of Pathology and Microbiology, College of Medicine University of Nebraska Medical Center Omaha Nebraska USA

**Keywords:** feeding groups, freshwater ecosystems, gut microbiome, macroinvertebrates

## Abstract

Despite their diversity, global distribution, and apparent effects on host biology, the rules of life that govern variation in microbiomes among host species remain unclear, particularly in freshwater organisms. In this study, we sought to assess whether geographic location, taxonomy (order, family, and genus), or functional feeding group (FFG) designations would best explain differences in the gut microbiome composition among macroinvertebrates sampled across 10 National Ecological Observatory Network's (NEON) freshwater stream sites in the United States. Subsequently, we compared the beta diversity of microbiomes among locations, taxonomy (order, family, and genus), and FFGs in a single statistical model to account for variation within the source microbial community and the types of macroinvertebrates sampled across locations. We determined significant differences in community composition among macroinvertebrate orders, families, genera, and FFGs. Differences in microbiome compositions were underscored by different bacterial ASVs that were differentially abundant among variables (four bacterial ASVs across the 10 NEON sites, 43 ASVs among the macroinvertebrate orders, and 18 bacterial ASVs differing among the five FFGs). Analyses of variations in microbiome composition using the Bray–Curtis distance matric revealed FFGs as the dominant source of variation (mean standard deviation of 0.8), followed by stream site (mean standard deviation of 0.5), and finally family and genus (mean standard deviation of 0.3 each). Our findings revealed a principal role for FFG classification in insect gut microbiome beta diversity with additional roles for geographic distribution and taxonomy.

## Graphical abstract

Model predictions for mean Bray–Curtis distances for stream locations, macroinvertebrate family, genus, and functional feeding group are assessed. The densities show the posterior distributions of Bray–Curtis distances for each level of stream, family, genus, and functional feeding group. The dot and error bars under each density show the median and 95% credible intervals. Tick marks represent the raw data.

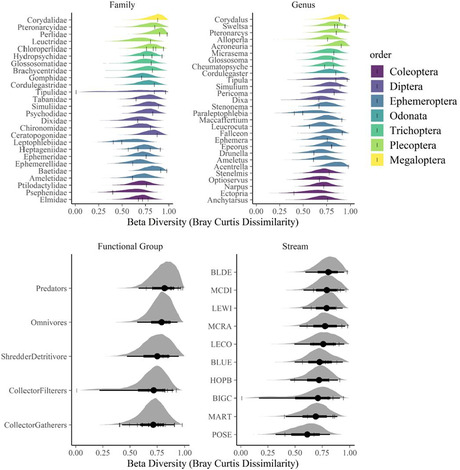

## 1 INTRODUCTION

A sizeable portion of the Earth's diversity occurs in the microbiomes, the micro‐organisms that live within host species (Hug et al., 2016; Louca et al., 2019). In studies of terrestrial insect gut microbiomes, stochastic and deterministic (at opposite ends of a spectrum) processes are essential to gut microbial assembly (Hanson et al., 2012; Jizhong & Daliang, 2017). Stochastic processes (or ecological processes) such as priority effects, dispersal limitation, and ecological drift have been determined to be responsible for variations in microbial community composition (or ecological processes [Jizhong & Daliang, 2017]). Similarly, various deterministic factors (non‐random, niche‐based mechanistic processes) also account for considerable variations in microbial community composition (free‐living and host‐associated). Deterministic processes mainly involve variation in environmental parameters (e.g., pH, salinity, DO, etc.), local habitat conditions, nutrient availability, and ultimately, co‐evolution between host and associated gut microbiomes (phylosymbiosis) (Jizhong & Daliang, 2017). These insights have generated various conceptual frameworks to assess the dynamics governing community assembly (Vellend, 2010) and by extension, gut microbial community assembly in insect hosts (Brown et al., 2020). The rules of life that govern variation in microbiomes among host species remain unclear, particularly in freshwater organisms.

In studies of freshwater invertebrate gut microbiomes, there are conflicting results regarding the importance of habitat, taxonomy (family and genus), and functional feeding group (FFG) categorization in shaping gut microbial assemblages of freshwater macroinvertebrates. One study determined the taxonomy to be a more relevant determinant of gut microbiome composition of freshwater macroinvertebrates than local habitat, stream conditions, or FFG (Kroetsch et al., 2020). In contrast, the vast majority of the very limited studies have determined FFGs (regardless of taxonomic affiliation of samples invertebrates) to be the principal variable explaining differences in microbiome composition across freshwater macroinvertebrates (Ayayee et al., 2018; Kaufman et al., 2000; Pechal & Benbow, 2016; Receveur et al., 2020). Interestingly, location has not been determined to be a significant determinant of freshwater macroinvertebrate gut microbiome composition in neither a study of samples collected from two streams in the same region (Ayayee et al., 2018) nor samples collected from multiple sites along the reach of one major river (Kroetsch et al., 2020).

A significant limitation of these prior studies of freshwater macroinvertebrate gut microbiomes has been the lack of systematic sampling of freshwater ecosystems at a broad scale. In addition, most of these studies only sampled multiple sites within single streams or limited streams (1¬3) within one geographic region. The novel aspect of this study is that it is the first to sample multiple streams at a large geographical scale. We assessed the gut microbiomes of insects collected from 10 National Ecological Observatory Network's (NEON) freshwater stream sites. Subsequently, in a single statistical model, we compared the beta diversity of microbiomes among locations, taxonomy (order, family, and genus), and FFGs. This allowed us to account for variation within the source microbial community and the types of macroinvertebrates sampled across locations. Microbial community compositions are expected to vary due to the underlying geology, land usage, and stream conditions of these NEON sites (Atashgahi et al., 2015; Drury et al., 2013; Fang et al., 2017; Hosen et al., 2017; Kaushal et al., 2021; Medeiros et al., 2016; Wakelin et al., 2008). Subsequently, microbial community compositions are also expected to vary among FFGs, since they are known to have differences in gut physiology (Anderson & Cargill, 1987; Austin & Baker, 1988; Cummins, 1979; Martin et al., 1980; Martin, Martin, et al., 1981; Tierno de Figueroa et al., 2011), and consequently, associated gut microbiomes (Ayayee et al., 2018; Pechal & Benbow, 2016; Receveur et al., 2020) and functions (Stief, 2013; Stief et al., 2009).

In this study, we sought to assess whether geographic location, taxonomy (order, family, and genus), or FFG designations would best explain differences in gut microbiome composition among sampled macroinvertebrates across 10 streams in North America. We hypothesized that differences in gut microbial community composition (β‐diversity) among sampled macroinvertebrates would be a function of deterministic factors (reflecting FFG designations) rather than stochastic factors (geographical location or taxonomical identification). This assumes that different macroinvertebrate taxa from different sites will have comparable gut physiologies, which will select for taxonomically comparable microbes, thus resulting in similar gut microbiomes.

## 2 MATERIALS AND METHODS

### 2.1 Sample collection

**FIGURE 1:**
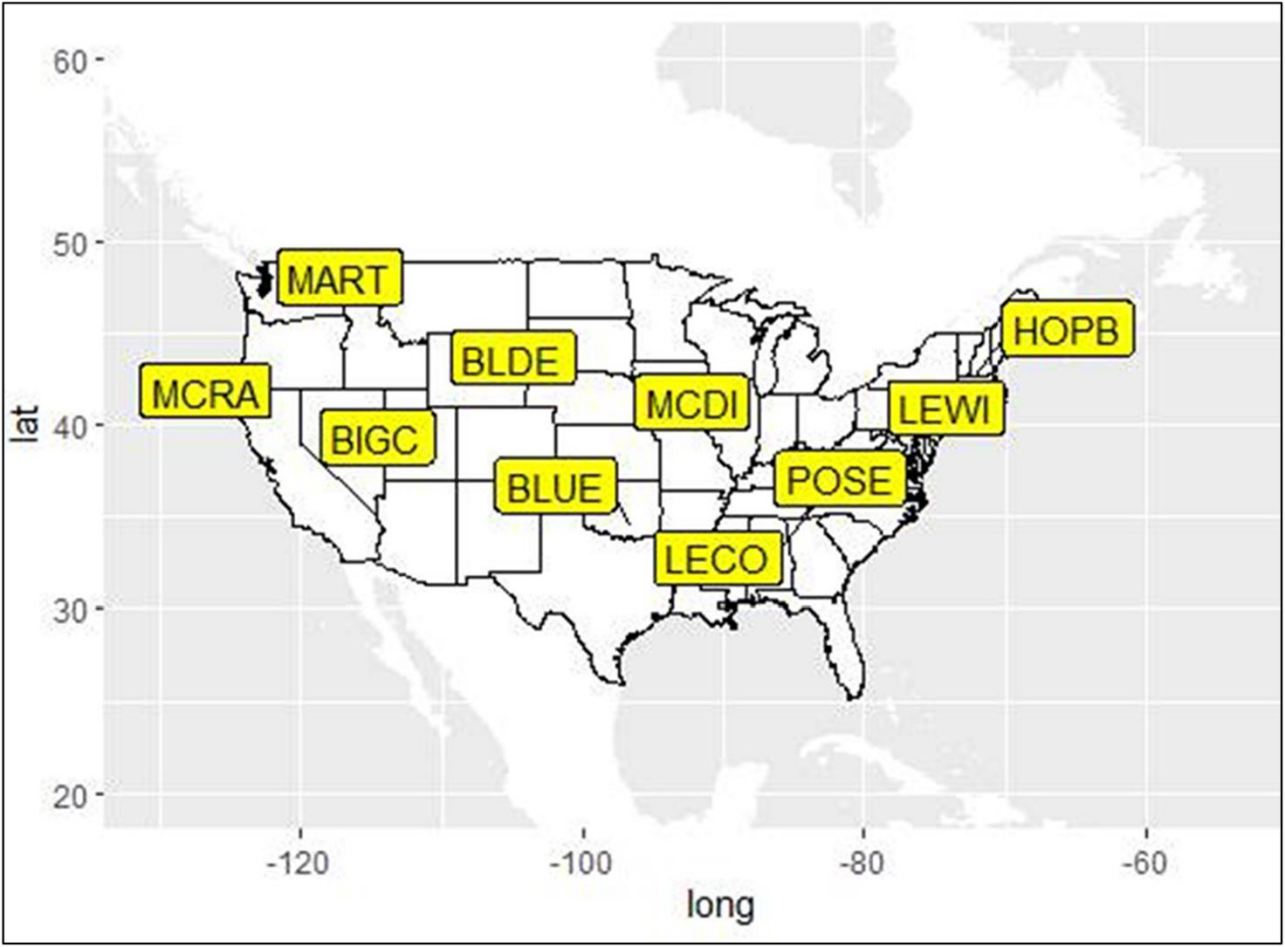
The distribution of the 10 NEON long‐term ecological research freshwater stream sampling sites across the United States.

Aquatic insect samples were obtained from collections acquired by the National Ecological Observatory Network (NEON) sites in the United States in 2020 (https://www.neonscience.org/field‐sites/about‐field‐sites) (NEON, 2022). We obtained macroinvertebrate samples collected from 10 NEON‐managed field sites across North America that ranged across 11 degrees of latitude with mean annual stream temperatures from 4.4 (BLDE) to 16°C (BLUE) (Figure 1). Samples were collected from each of the 10 sites by NEON teams using conventional Surber samplers and D‐frame nets (Parker, 2022). At eight of the 10 sites, samples were collected in the fall (September, October, or November). At the remaining two sites (LEWI and MCDI), samples were collected in spring (March or June). Due to permit restrictions, we limited sample selection from NEON collections to five individuals per taxon per site. According to NEON protocols, macroinvertebrate samples were identified and verified to both species and family taxonomic levels (NEON, 2022; Parker, 2022) using published field guides and keys (Cummins, 2021; Merritt et al., 2008). The insect samples were stored in 1.5 ml tubes in 80% ethanol and stored at −20°C until DNA extraction. Upon receipt, insect samples were categorized into functional feeding groups using a combination of published (Cummins, 2021; Merritt et al., 2008) and online resources (https://www.macroinvertebrates.org/).

### 2.2 Sample processing, DNA extraction, and Illumina sequencing

Before DNA extraction, we surface‐sterilized insect samples by washing them in a 1% detergent solution (Micro‐90, Capitol Scientific, Inc) for 1 min, followed by two 1‐min rinses in deionized water (DI) water. Additionally, we used the diluted detergent to surface sterilize the outside of the insect samples prior to any tissue homogenization for DNA extraction. Finally, the DNA quantity and quality both in‐house and at the sequencing center indicated that using the diluted detergent did not affect the stability and structure of the DNA obtained from the insect samples it was used to sterilize. A previous study found no effect of storage or surface sterilization methods on gut bacterial community assessments (Hammer et al., 2015). If samples were large enough, then we dissected the entire dietary system; if not, we used the whole insect. Briefly, dissections were performed in a designated clean area. The dissecting tools were maintained in a 10% bleach solution during the entire dissection process and were rinsed in DI water every time before usage. The entire process of surface sterilizing, dissection, and DNA extraction was performed using gloves.

Next, we performed DNA extraction using the DNeasy Blood & Tissue Kit (Qiagen, Germantown, MD, USA) with modifications to the manufacturer's directions. We verified the presence of microbial 16 S rRNA marker gene in all extracted DNA samples via PCR using the universal 27F and 1492R bacterial primer pair (Frank et al., 2008). Samples were submitted for high‐throughput paired‐end MiSeq library preparation and sequencing at the University of Nebraska Medical Center Genomics Core. Briefly, a limited cycle PCR reaction was performed on each sample to create a single amplicon, including the V4 (515‐F) and V5 (907‐R) variable region (Keskitalo et al., 2017). The resulting libraries were validated using the Agilent BioAnalyzer 2100 DNA 1000 chip (Agilent), and DNA was quantified using Qubit 3.0 (Qubit™, Thermofisher). A pool of libraries was loaded into the Illumina MiSeq at 10 pM. The pool was spiked with 25% PhiX (a bacteriophage) at 10 pM for MiSeq run quality as an internal control (Mukherjee et al., 2015) to generate 300 bp paired ends with the 600‐cycle kit (version 3). The raw reads were deposited into the Sequence Read Archive database (Accession number: PRJNA825559).

### 2.3 Microbiome data processing and statistical analyses

Acquired fastq primer‐trimmed MiSeq paired‐end reads from the sequencing center were processed using DADA2 (Callahan et al., 2016). Across both forward and reverse reads, filtering excluded reads with more than two expected erroneous base calls, any reads identified as part of the PhiX bacteriophage genome for quality control, and reads less than 175 base pairs. Forward reads were truncated to 250 base pairs, and reverse reads to 200 base pairs. Truncation was performed to maintain median quality scores above 30 across samples. Reads were merged, and chimeras were subsequently filtered out. We determined amplicon sequence variants (ASVs) and representative sequences against the SILVA 138.1 16 S rRNA gene reference database (Quast et al., 2012). We combined the count and taxonomy information for the generated ASVs into a classical ASV table, and further analyses were performed in QIIME v.1.8 (Caporaso et al., 2010; Kuczynski et al., 2012). Before analyses, we curated the table by removing unclassified reads at the bacterial or archaeal domain level, and any reads assigned as Eukaryotes. Finally, samples with less than 1000 reads per sample were removed from the table before analyses. We then summarized the filtered and curated ASV table to the family level (García‐López et al., 2020, 2021), and all subsequent analyses were performed on this table.

Briefly, we rarefied the family‐level table to 1110 reads per sample and replicated 10 times across all samples. The rationale and justification for rarefying have been discussed in prior studies (Cameron et al., 2021; McKnight et al., 2019; Weiss et al., 2017). For alpha diversity, the chao1 (Huang & Zhang, 2013), Shannon's evenness (Shannon, 1957), and observed OTUs indices were calculated in QIIME, and significant differences among categorical groupings were determined via non‐parametric Wilcoxon tests in JMP Pro 15 (S.A.S.). We generated the Bray–Curtis dissimilarity distance matrix (Bray & Curtis, 1957) using the 1110‐rarefied table. The rationale for choosing the Bray–Curtis distance matrix has been previously outlined (Anderson et al., 2011). The calculated distance matrix was used to calculate the non‐metric multidimensional scales (NMDS) in QIIME. The NMDS scales are used to visualize categorical sample groupings that differ in microbiome composition following a test of differences among these categorical groupings via permutational multivariate analysis of variance (PERMANOVA) (Anderson, 2017) in QIIME using the compare_categories.py command. We performed an indicator species analysis on the ASV table using the categories (insect order, FFG, location, etc.) to examine microbial community members most likely driving differences among microbiomes using the group significance command in QIIME (version 1.9) followed by a Kruskal–Wallis test. Figures were generated using JMP Pro 14 (SAS).

### 2.4 Modeling of factors and microbiome composition

We compared the influence of freshwater insect taxonomy (orders, family, and genus), functional feeding groups, and geographical (i.e., NEON) sites on the beta diversity of associated gut microbiomes. To do this, we used intercept‐only random effects models with the response variable (Bray–Curtis distance) as a measure of diversity and a Beta likelihood with a logit link. Each model contained a response (grand mean) and three varying intercepts (insect order [family and genus nested within this], functional feeding group, and NEON site), also known as random effects (McElreath, 2020). The structure of the model is as follows:

$$y_{i}∼\text{Beta}\;\left(\mu ∙\varphi ,\left(1-\mu \right)∙\varphi \right)$$

$$\text{logit}\left(\mu \right)=\alpha +\alpha_{\text{Family}}+\alpha_{\text{Genus}}+\alpha_{\mathrm{FFG}}+\alpha_{\text{Site}}$$

$$\;\alpha ∼\text{Normal}\left(0,1\right)$$

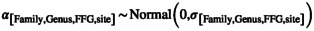

$$\sigma_{\left[\text{Family},\text{Genus},\mathrm{FFG},\text{site}\right]}∼\text{Exponential}\left(0.5\right)$$

$$\varphi ∼\text{Gamma}\left(0.01,0.01\right)$$

Where $y_{i}$ is the Bray–Curtis value of the *i*th observation arising from a beta distribution with parameters alpha = μ∙φ and beta = $\left(1-\mu \right)∙\varphi$. In this parameterization, the mean is the primary target of inference and is represented by μ, while φ is a scalar. In addition to this model, we used model comparison (Hooten & Hobbs, 2015) to determine which predictor variable best explained beta diversity. We fit four models, each with a single fixed predictor of family, genus, functional feeding group, or stream site. The remaining predictors (i.e., those not specified as fixed effects) were included as varying intercepts. We then compared these models using the Watanabe‐Akaike Information Criterion (WAIC) (Hooten & Hobbs, 2015). Priors for each parameter were chosen using prior predictive simulation (Wesner & Pomeranz, 2021) and are justified in the Appendix S1.

There are several advantages to this modeling approach (Dietze, 2017). First, the varying intercepts use partial pooling to pull each group's mean (i.e., mean of individual orders or FFGs or sites, etc.) toward the global mean. That provides conservative estimates of Bray–Curtis values for each group because the amount of pooling is determined by the amount of data in each group. In other words, it provides a correction for outliers such that groups with few data points are treated skeptically and pulled more strongly toward the overall mean (Efron & Morris, 1977). This is especially important for data like ours with relatively low replication within each group, helping to prevent spurious conclusions. Second, the residual variance of the grand mean of Bray–Curtis dissimilarity is partitioned among the varying intercepts. This allows us to identify the variables that contribute most of the variation in beta diversity, which are likely to be the best variables to focus on in future studies. Third, from these models, we can predict the diversity of the gut microbiomes at each level (e.g., for each species, stream, or functional feeding group). Fourth, the varying intercepts allow predictions, with proper uncertainty, of the microbiomes of insects that are not currently in our dataset (McElreath, 2020). Finally, varying intercepts automatically adjust for unbalanced data so that no single sample dominates the inference.

We fit the model using Bayesian inference in R version 4.2.0 (R Core Team, 2022), with the *brms* package (Bürkner, 2017). Posteriors were explored in *rstan* (Stan Development Team, 2022) with Hamiltonian Monte Carlo (No‐U‐Turn sampler). We used four chains with 2000 iterations each, and the first 1000 were discarded as a warmup. Model convergence was checked by ensuring that all R‐hats were <1.1 and visually assessing the chains for mixing.

## 3 RESULTS

### 3.1 Macroinvertebrate summary

**FIGURE 2:**
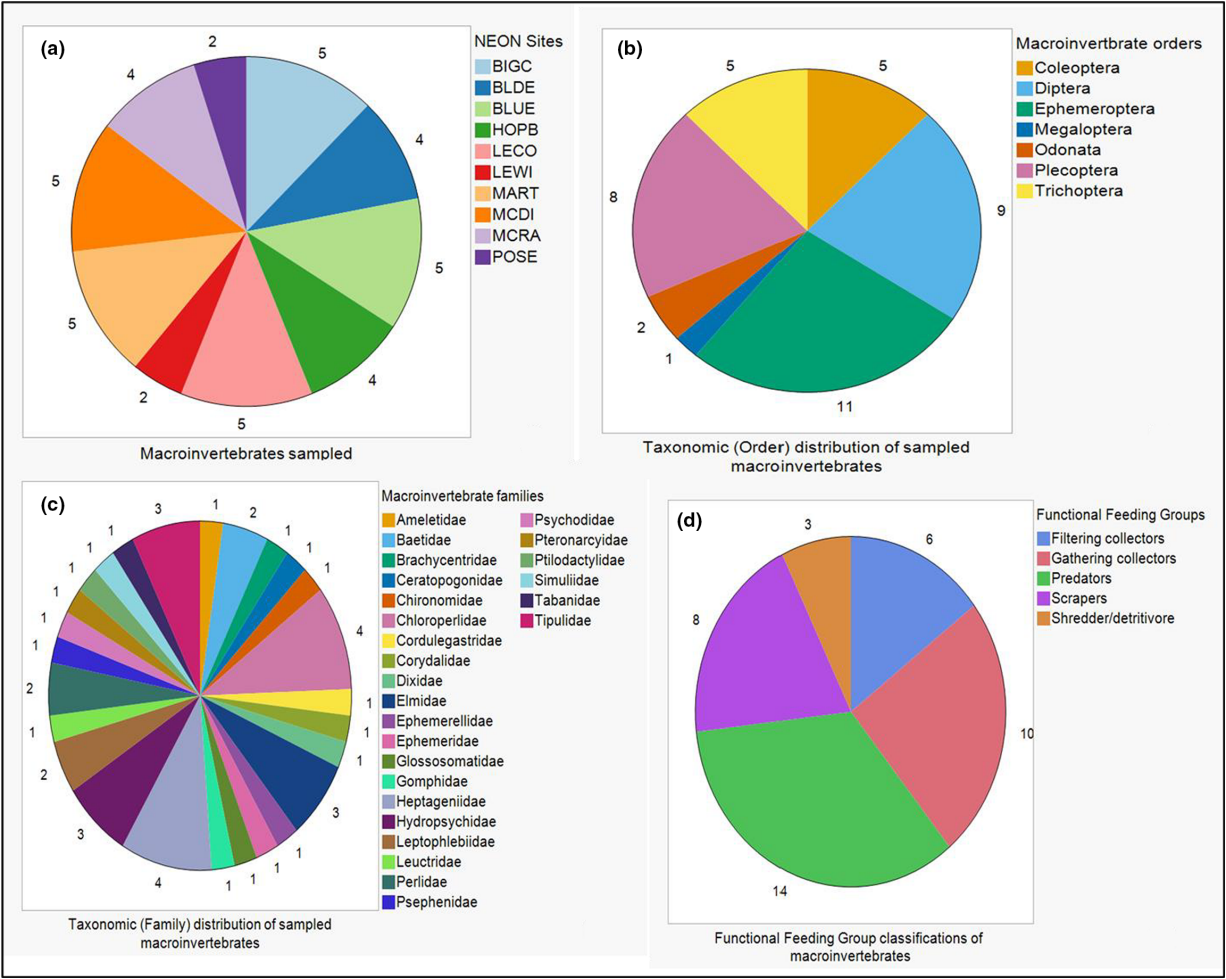
Macroinvertebrate summary. The breakdown of freshwater macroinvertebrates was obtained from the 10 NEON sites, seven macroinvertebrate orders, 26 families, and five functional feeding group designations used in this study.

We obtained 45 macroinvertebrate samples from 10 NEON sites across the continental USA, with each sample representing one to five individuals from a single genus or family. After removing four samples that did not meet sequence quality thresholds, our final dataset consisted of 41 samples (Table 1A). Taxonomically, the 41 macroinvertebrate samples represented seven orders (Figure 2b), 26 families (Figure 2c), and 36 genera across all 10 NEON sites. The major orders in the dataset were Ephemeroptera (*n* = 11 samples), Diptera (*n* = 9 samples), and Plecoptera (*n* = 8 samples) (Figure 2b). The major families were Heptageniidae (*n* = 4 samples) and Chloroperlidae (*n* = 4 samples), followed by Elmidae, Hydropsychidae, and Tipulidae (all *n* = 3 samples) (Figure 2c). We further classified the 41 macroinvertebrate samples into four FFGs: filtering collectors, gathering collectors, predators, and shredder/detritivores (Figure 2d). Each FFG contained between 2 and 17 genera and between 1 and 11 families. The taxonomic and ecological information of the 41 macroinvertebrate samples used in this study are provided in Tables 1A and 1B.

**TABLE 1A:** Summary of the macroinvertebrate samples obtained and used in this study.

Sample ID	Order	Family	taxon name	NEON locations	Genera	Functional feeding group
LEWI‐A1	Diptera	Ceratopogonidae	Ceratopogoninae sp.	LEWI	*NA*	Predators
LEWI‐A2	Diptera	Tabanidae	Tabanidae sp.	LEWI	*NA*	Predators
MCRA‐A11	Ephemeroptera	Heptageniidae	Epeorus sp.	MCRA	*Epeorus*	Scrapers
MCRA‐A12	Plecoptera	Perlidae	Perlidae sp.	MCRA	*NA*	Predators
MCRA‐A13	Trichoptera	Hydropsychidae	Arctopsychinae sp.	MCRA	*NA*	Filtering collectors
MCRA‐A15	Ephemeroptera	Ameletidae	Ameletus sp.	MCRA	*Ameletus*	Gathering collectors
BLUE‐A21	Diptera	Chironomidae	Chironomini sp.	BLUE	*NA*	Filtering collectors
BLUE‐A22	Coleoptera	Elmidae	Stenelmis sp.	BLUE	*Stenelmis*	Gathering collectors
BLUE‐A23	Ephemeroptera	Ephemeridae	Ephemera sp.	BLUE	*Ephemera*	Scrapers
BLUE‐A24	Ephemeroptera	Heptageniidae	Leucrocuta sp.	BLUE	*Leucrocuta*	Scrapers
BLUE‐A25	Megaloptera	Corydalidae	Corydalus sp.	BLUE	*Corydalus*	Predators
MCDI‐A31	Diptera	Simuliidae	Simulium sp.	MCDI	*Simulium*	Filtering collectors
MCDI‐A32	Diptera	Tipulidae	Tipula sp.	MCDI	*Tipula*	Predators
MCDI‐A33	Ephemeroptera	Heptageniidae	Stenonema femoratum	MCDI	*Stenonema*	Scrapers
MCDI‐A34	Trichoptera	Hydropsychidae	Cheumatopsyche sp.	MCDI	*Cheumatopsyche*	Filtering collectors
MCDI‐A35	Ephemeroptera	Baetidae	Fallceon sp.	MCDI	*Fallceon*	Gathering collectors
HOPB‐A41	Trichoptera	Hydropsychidae	Cheumatopsyche sp.	HOPB	*Cheumatopsyche*	Filtering collectors
HOPB‐A42	Trichoptera	Glossosomatidae	Glossosoma sp.	HOPB	*Glossosoma*	Scrapers
HOPB‐A43	Ephemeroptera	Heptageniidae	Maccaffertium sp.	HOPB	*Maccaffertium*	Scrapers
HOPB‐A44	Ephemeroptera	Leptophlebiidae	Paraleptophlebia sp.	HOPB	*Paraleptophlebia*	Gathering collectors
BLDE‐A51	Trichoptera	Brachycentridae	Micrasema sp.	BLDE	*Micrasema*	Gathering collectors
BLD3‐A52	Ephemeroptera	Baetidae	Acentrella sp.	BLDE	*Acentrella*	Gathering collectors
BLDE‐A53	Diptera	Psychodidae	Pericoma/Telmatoscopus sp.	BLDE	*Pericoma*	Gathering collectors
BLDE‐A54	Plecoptera	Chloroperlidae	Sweltsa sp.	BLDE	*Sweltsa*	Predators
LECO‐A61	Diptera	Dixidae	Dixa sp.	LECO	*Dixa*	Filtering collectors
LECO‐A62	Coleoptera	Ptilodactylidae	Anchytarsus bicolor	LECO	*Anchytarsus*	Shredder/detritivore
LECO‐A63	Plecoptera	Pteronarcyidae	Pteronarcys sp.	LECO	*Pteronarcys*	Shredder/detritivore
LECO‐A64	Plecoptera	Perlidae	Acroneuria sp.	LECO	*Acroneuria*	Predators
LECO‐A65	Plecoptera	Chloroperlidae	Alloperla sp.	LECO	*Alloperla*	Predators
BIGC‐A71	Diptera	Tipulidae	Tipulidae sp.	BIGC	*NA*	Predators
BIGC‐A72	Odonata	Cordulegastridae	Cordulegaster sp.	BIGC	*Cordulegaster*	Predators
BIGC‐A73	Odonata	Gomphidae	Gomphidae sp.	BIGC	*NA*	Predators
BIGC‐A74	Plecoptera	Chloroperlidae	Sweltsa sp.	BIGC	*Sweltsa*	Predators
BIGC‐A75	Plecoptera	Leuctridae	Leuctridae sp.	BIGC	*NA*	Shredder/detritivore
MART‐B1	Diptera	Tipulidae	Tipula sp.	MART	*Tipula*	Predators
MART‐B2	Plecoptera	Chloroperlidae	Sweltsa sp.	MART	*Sweltsa*	Predators
MART‐B3	Ephemeroptera	Ephemerellidae	Drunella doddsii	MART	*Drunella*	Scrapers
MART‐B4	Coleoptera	Elmidae	Narpus sp.	MART	*Narpus*	Gathering collectors
MART‐B5	Coleoptera	Psephenidae	Ectopria sp.	MART	*Ectopria*	Scrapers
POSE‐B11	Coleoptera	Elmidae	Optioservus sp.	POSE	*Optioservus*	Gathering collectors
POSE‐B12	Ephemeroptera	Leptophlebiidae	Paraleptophlebia sp.	POSE	*Paraleptophlebia*	Gathering collectors

**TABLE 1B:** Summary of the macroinvertebrate samples obtained and used in subset analyses.

Sample ID	Locations	Family	Feeding group	Order	Genus taxized
MCDI‐A32	MCDI	Tipulidae	Predators	Diptera	Tipula
HOPB‐A44	HOPB	Leptophlebiidae	Gathering collectors	Ephemeroptera	Paraleptophlebia
BLDE‐A54	BLDE	Chloroperlidae	Predators	Plecoptera	Sweltsa
BIGC‐A74	BIGC	Chloroperlidae	Predators	Plecoptera	Sweltsa
MART‐B1	MART	Tipulidae	Predators	Diptera	Tipula
MART‐B2	MART	Chloroperlidae	Predators	Plecoptera	Sweltsa
POSE‐B12	POSE	Leptophlebiidae	Gathering collectors	Ephemeroptera	Paraleptophlebia

### 3.2 Freshwater macroinvertebrate gut microbiome diversity and composition

Overall, we obtained ~6.5 million reads from the sequencing effort. Filtering, merging, chimera removal, and curating of the resulting count and taxonomy table (removal of unassigned reads at the domain level and removal of samples with fewer than 1000 reads per sample) resulted in ~86% of reads retained (5,684,379 reads). These were distributed across 41 samples yielding 12,658 ASVs (mean reads per sample = 19,468; minimum: 1235.000, maximum: 98,479.000). Rarefaction curves for the species diversity and richness indices at ~1110 reads per sample indicate that most microbial diversity had been sufficiently covered across samples (Figure S1). There were no significant differences among locations, taxonomy (order, family, genera), or FFGs for all four diversity indices evaluated (Table 2). Further investigation of microbial community composition (β‐diversity) among macroinvertebrate samples depended on the inquiry level.

**TABLE 2:** Non‐parametric test output of alpha diversity metrics for macroinvertebrate sample variables (location, order, family, genera, and functional feeding group (FFGs).

		Diversity indices	
Variables	Observed species	Chao1	Shannon's
Location	*χ* ^2^ = 12.76, *p* = .17	*χ* ^2^ = 12.61, *p* = .18	*χ* ^2^ = 11.61, *p* = .24
Order	*χ* ^2^ = 8.10, *p* = .23	*χ* ^2^ = 5.37, *p* = .49	*χ* ^2^ = 3.67, *p* = .72
Family	*χ* ^2^ = 27.71, *p* = .32	*χ* ^2^ = 23.03, *p* = .57	*χ* ^2^ = 27.98, *p* = .31
Genus	*χ* ^2^ = 34.90, *p* = .52	*χ* ^2^ = 34.18, *p* = .55	*χ* ^2^ = 37.02 *p* = .42
Functional feeding group	*χ* ^2^ = 5.74, *p* = .22	*χ* ^2^ = 3.92, *p* = .42	*χ* ^2^ = 9.15, *p* = .057

**FIGURE 3:**
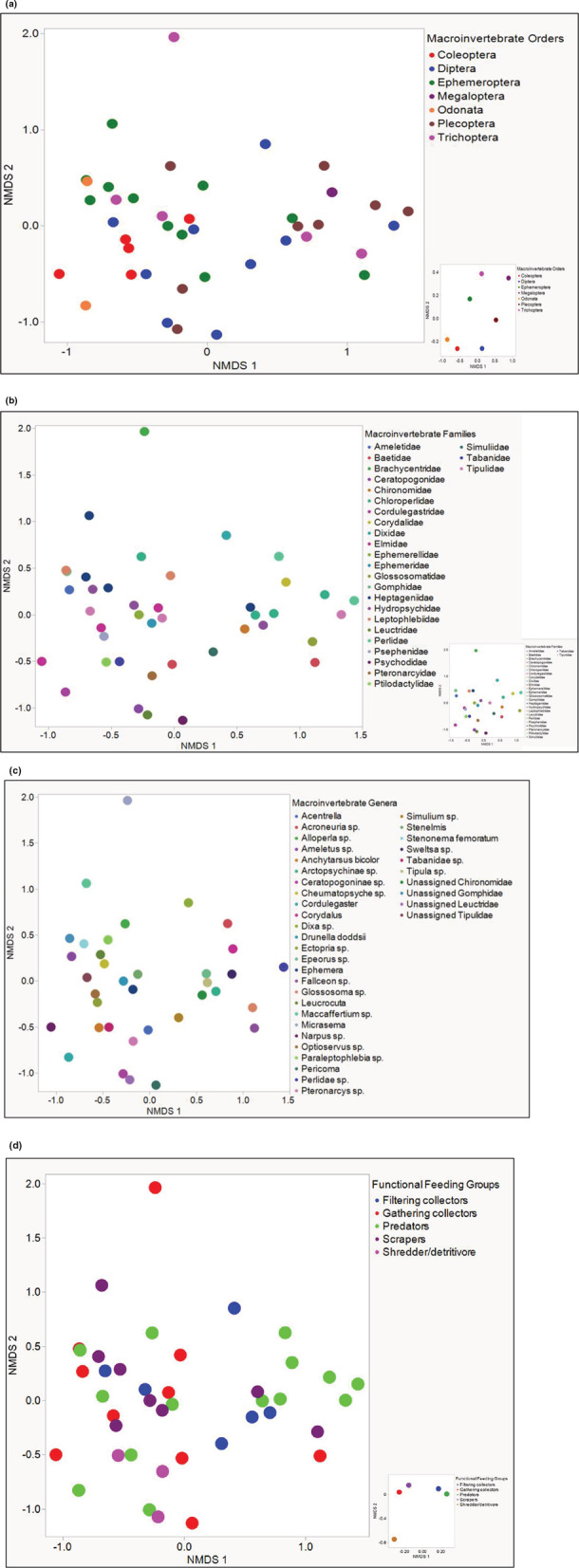
Gut microbial composition among macroinvertebrate orders (a). Gut microbial composition among macroinvertebrate families (b). Gut microbial composition among macroinvertebrate genera (c). Gut microbial composition among macroinvertebrate functional feeding groups (d). Gut microbial composition among macroinvertebrate sample clusters (e).

Overall, variances did not differ significantly among samples for locations (PERMDISP: *F* = 1.38, *p* = .3), order (PERMDISP: *F* = 1.97, *p* = .09), family (PERMDISP: *F* = 1.83, *p* = .11), and FFGs (PERMDISP: *F* = 1.6, *p* = .2). There were significant variances in genus (PERMDISP: *F* = 4.4428e+30, *p* < .001). Across locations, there were no significant differences among the 10 NEON sites (PERMANOVA: test statistic = 1.13, *p* = .15). However, there were significant differences in community composition among macroinvertebrate orders (PERMANOVA: test statistic = 1.76, *p* < .001) (Figure 3a), families (PERMANOVA: test statistic = 1.50, *p* < .001) (Figure 3b), and genera (PERMANOVA: test statistic = 1.89, *p* = .004) (Figure 3c). Finally, there was a significant difference in microbiome composition among ecological functional feeding group classifications (PERMANOVA: test statistic = 1.58, *p* = .008) (Figure 3d).

**FIGURE 4:**
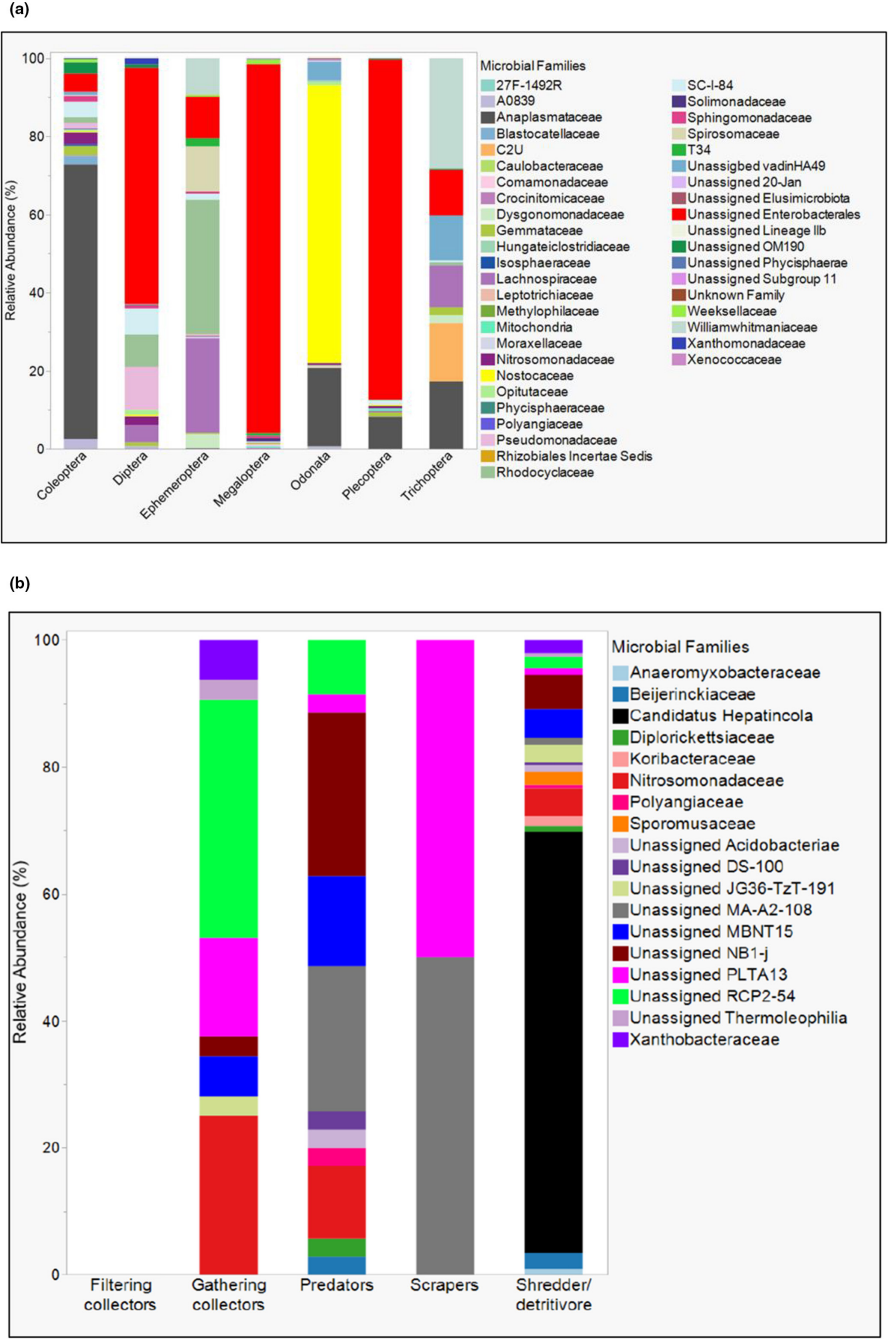
Relative abundance of differentially abundant bacterial ASVs at the family level that may be underscoring differences in microbiome community composition among freshwater macroinvertebrate orders (a) and functional feeding groups (FFGs) (b) used in this study.

Examination of ASVs that differed significantly in abundance among the categories studied (location, macroinvertebrate order, and FFG) yielded four bacterial ASVs across the 10 NEON sites (Kruskal–Wallis test; FDR‐adjusted *p* = .05). All four significantly abundant ASVs were only detected in one NEON site (LEWI). These were ASVs classified into the families Bacteroidetes vadinHA17 (32%), Bacteroidetes BD2‐2 (28%), *Defluviicoccaceae* (28%), and *Geobacteraceae* (12%). There were 43 ASVs that differed among the macroinvertebrate orders (Kruskal–Wallis test; FDR‐adjusted *p* = .05) (Figure 4a). Finally, for FFGs, 18 bacterial ASVs differed among the five FFGs (Kruskal–Wallis test; FDR‐adjusted *p* = .05) (Figure 4b).

Among macroinvertebrate orders, *Anaplasmataceae* (Genus *Wolbachia*) (abundant across five macroinvertebrate orders except for Diptera and Megaloptera) and Unassigned Enterobacterales (abundant across six macroinvertebrate orders, except Odonata) were the most abundant ASVs. *Anaplasmataceae* (Genus *Wolbachia*) was the most abundant in Coleoptera (70.28%), followed by Odonata (20.08%) and Trichoptera (17.25%) (Figure 4a). Clustering of Odonata and Coleoptera together is underscored by the abundance of *Anaplasmataceae* (Genus *Wolbachia*) in these orders. Odonata is further separated from Coleoptera due to the abundance of *Nostocaceae* (71.1%) and unassigned vadinHA49 (4.83%) in this Odonata and the presence of several other ASVs only significantly abundant in Coleoptera (Figure 4a). The clustering of the Megalopteran samples away from the other macroinvertebrate orders is underscored by the dominance of unassigned Enterobacterales (94.4%). In contrast, the clustering of the Plecopteran samples from the other macroinvertebrate orders is underscored by the dominance of both unassigned Enterobacterales (87.1%) and *Anaplasmataceae* (8.2%) (Figure 4a). The comparatively higher abundances of *Lachnospiraceae* (24.2%), *Rhodocyclaceae* (34.4%), *Spirosomaceae* (11.57%), and unassigned Enterobacterales (10.64%) separate the Ephemeroptera from the Diptera and other orders. Among functional feeding groups, the separate clustering of the shredder/detritivore group from the other four functional feeding groups (Figure 3d) is underscored by the preponderance of bacterial ASVs mainly in the family *Candidatus Hepatincola* (order Rickettsiales) (63.5%), as well as a more diverse representation of ASVs that differed in abundances in this functional feeding group (Figure 4b). The clustering of the filtering collectors and predator (Figure 3d) is underscored by the abundances of *Nitrosomonadaceae* (26.19%), unassigned B1‐j (21.43%), unassigned MA‐A2‐108 (19.04%), unassigned MBNT15 (11.9%), and unassigned RCP2‐54 (7.1%), as well as unassigned DS‐100, unassigned Acidobacteria, and *Polyangiaceae* (all 2.38%) predominantly in predators. Filtering collectors had no bacterial ASVs that were significantly abundant across functional feeding groups. The clustering together of gathering collectors and scrapers separate from the other functional feeding groups (Figure 3d) can be attributed to the relatively higher proportional abundances of unassigned PLTA13 (10.87% in gathering collectors and 50% in scrapers) in both groups (Figure 4b); Gathering collectors are further characterized by the abundances of *Nitrosomonadaceae* (47.8%), unassigned RCP2‐54 (26.08%), unassigned MBNT15 and *Xanthobacteraceae* (*both 4.38%*), and unassigned Thermolephilia (2.17%). In contrast, the scrapers are characterized by the abundance of unassigned MA‐A2‐108 (50%) (Figure 4b).

An unexpected but intriguing result from our microbiome analyses was the detection of the endosymbiont bacterium, *Wolbachia*, (family *Anaplasmataceae*) in analyzed freshwater macroinvertebrate orders (Figure 4a). *Wolbachia* was most abundant in aquatic Coleoptera (70.28%), followed by Odonata (20.08%), Trichoptera (17.28%), Plecoptera (8.20%), and Ephemeroptera (0.15%). This bacterial ASV was not detected in aquatic Diptera and Megaloptera macroinvertebrate orders. A subsequent PCR‐based investigation of the presence of *Wolbachia* in the macroinvertebrate samples using *wsp* primers with modifications to reported conditions (Sazama et al., 2017) resulted in six positive detections and 41 negatives (Table 3). The six positive results are distributed across four macroinvertebrate orders, Coleoptera (Family *Elmidae*), Odonata (Family *Gomphidae* and *Cordulegastridae*), Trichoptera (Family *Glossosomatidae*), and Plecoptera (Family *Chloroperlidae*) (Table 3).

**TABLE 3:** Results of *wsp*‐PCR on freshwater macroinvertebrates obtained from 10 NEON freshwater long‐term ecological research sites.

Sample ID	Order	Family
LEWI‐A1	Diptera	Ceratopogonidae
LEWI‐A2	Diptera	Tabanidae
MCRA‐A11	Ephemeroptera	Heptageniidae
MCRA‐A12	Plecoptera	Perlidae
MCRA‐A13	Trichoptera	Hydropsychidae
MCRA‐A15	Ephemeroptera	Ameletidae
BLUE‐A21	Diptera	Chironomidae
***BLUE‐A22***	***Coleoptera***	***Elmidae***
BLUE‐A23	Ephemeroptera	Ephemeridae
BLUE‐A24	Ephemeroptera	Heptageniidae
BLUE‐A25	Megaloptera	Corydalidae
MCDI‐A31	Diptera	Simuliidae
MCDI‐A32	Diptera	Tipulidae
MCDI‐A33	Ephemeroptera	Heptageniidae
MCDI‐A34	Trichoptera	Hydropsychidae
MCDI‐A35	Ephemeroptera	Baetidae
HOPB‐A41	Trichoptera	Hydropsychidae
***HOPB‐A42***	***Trichoptera***	***Glossosomatidae***
HOPB‐A43	Ephemeroptera	Heptageniidae
HOPB‐A44	Ephemeroptera	Leptophlebiidae
BLDE‐A51	Trichoptera	Brachycentridae
BLD3‐A52	Ephemeroptera	Baetidae
BLDE‐A53	Diptera	Psychodidae
***BLDE‐A54***	***Plecoptera***	***Chloroperlidae***
LECO‐A61	Diptera	Dixidae
LECO‐A62	Coleoptera	Ptilodactylidae
LECO‐A63	Plecoptera	Pteronarcyidae
LECO‐A64	Plecoptera	Perlidae
LECO‐A65	Plecoptera	Chloroperlidae
BIGC‐A71	Diptera	Tipulidae
***BIGC‐A72***	***Odonata***	***Cordulegastridae***
***BIGC‐A73***	***Odonata***	***Gomphidae***
BIGC‐A74	Plecoptera	Chloroperlidae
BIGC‐A75	Plecoptera	Leuctridae
MART‐B1	Diptera	Tipulidae
MART‐B2	Plecoptera	Chloroperlidae
MART‐B3	Ephemeroptera	Ephemerellidae
MART‐B4	Coleoptera	Elmidae
MART‐B5	Coleoptera	Psephenidae
**POSE‐B11**	**Coleoptera**	**Elmidae**
POSE‐B12	Ephemeroptera	Leptophlebiidae

*Note*: Positive samples are highlighted in bold.

### 3.3 Assessment of variables shaping gut microbiomes

**FIGURE 5:**
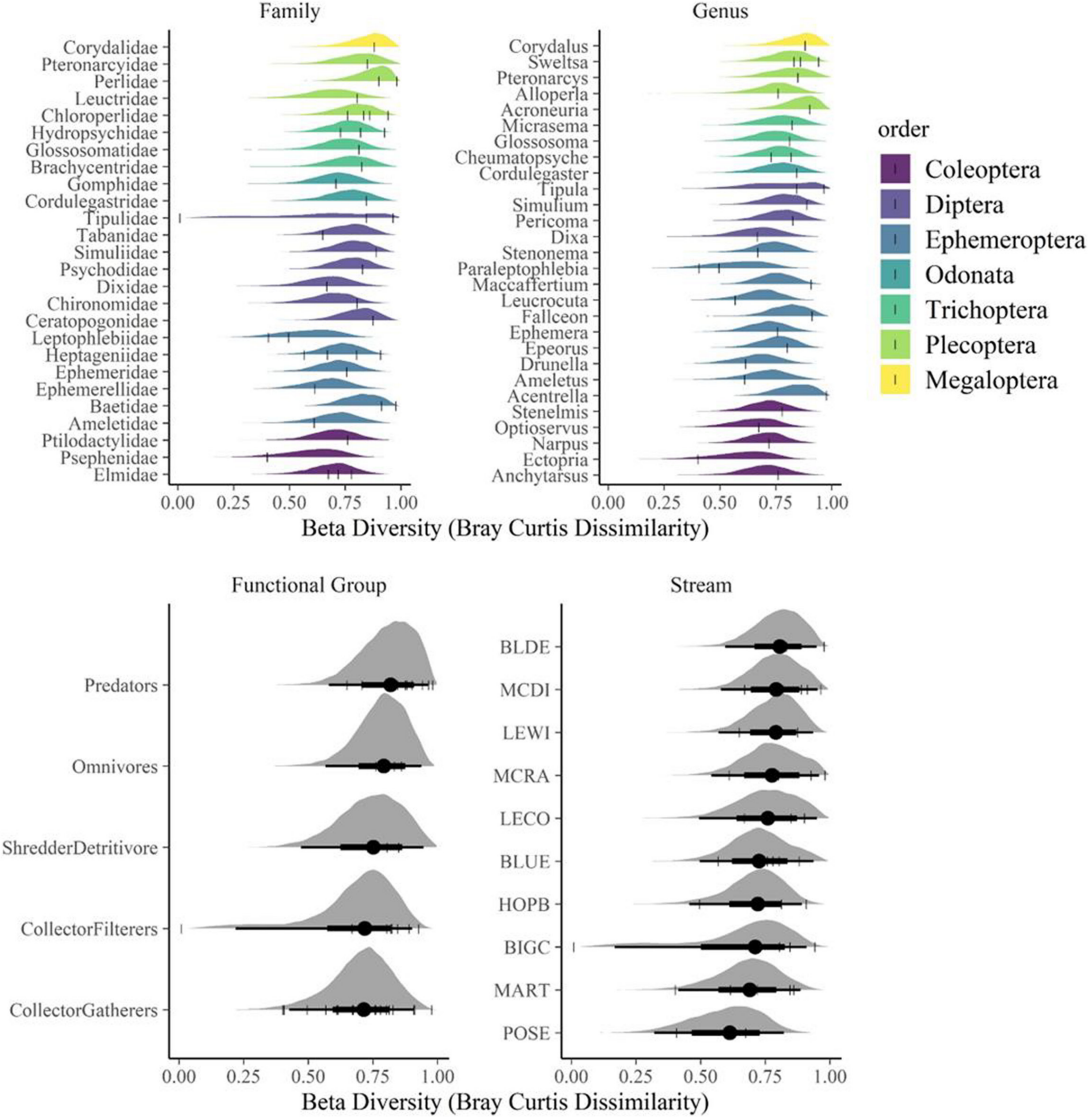
Model results for beta diversity as measured by Bray–Curtis distances. Model predictions for mean Bray–Curtis distances for stream locations, macroinvertebrate family, genus, and functional feeding group. The densities show the posterior distributions of Bray–Curtis distances for each level of stream, family, genus, and functional feeding group. The dot and error bars under each density show the median and 95% credible intervals. Tick marks represent the raw data.

Among all samples, beta diversity (in units of Bray–Curtis distance) averaged ~0.7 ± 0.09 (posterior mean ± SD) (Figure 5a). Among families, beta diversity ranged from a mean of 0.5 (95% credible interval: 0.3–0.8) in Leptophlebiidae (represented by a single genus, Paraleptophlebia) to 0.9 (0.69–0.97) in Perlidae (represented by *Acroneuria* and an unknown genus) (Figure 5a). Among FFGs, beta diversity ranged from 0.7 (0.2–0.9) in collector/filterers to 0.8 in predators (0.6–0.9) and among sites, it ranged from 0.6 (0.3–0.8) at POSE to 0.8 (0.6–0.95) at BLDE (Figure 5a). The model selection procedure indicated that no model dominated in explaining beta diversity (Table 4). For example, the standard error of delta WAIC overlapped for the top three models (genus, family, and FFG), as did the standard errors of *R* ^2^ (Table 4). In addition, no model had a mean of *R* ^2^ that was >0.5. This indicates that no single factor was dominant in explaining microbiome beta diversity in aquatic insects.

**TABLE 4:** Model selection results for four candidate models predicting microbiome beta diversity.

Fixed	Random	WAIC_delta_	SE(WAIC_delta_)	R^2^	SE(R^2^)
Genus	(Fam, FFG, Site)	0.0	0.0	0.54	0.15
Family	(Gen, FFG, Site)	0.7	1.4	0.51	0.14
Functional Group	(Gen, Fam, Site)	5.5	5.8	0.40	0.18
Stream site	(Gen, Fam, FFG)	9.3	6.4	0.37	0.17

*Note*: Each model contains one fixed factor and three random effects. WAIC_delta_ is the difference in WAIC scores between the best performing model and each subsequent model (along with the standard error of those differences). *R* ^2^ is the Bayesian version of standard *R* ^2^, along with its standard error.

## 4 DISCUSSION

The nature and dynamics of insect–gut microbial associations are well established and understood for terrestrial insects (Dillon & Dillon, 2004; Douglas, 2015; Yun et al., 2014). For instance, there is a consensus on the impacts of diet, developmental stage, and environment on the gut microbiomes of terrestrial insects. However, such consensus is lacking for aquatic insects and their associated gut microbiomes. For example, the gut microbiome of freshwater macroinvertebrates has been noted to differ from the surrounding environment under controlled laboratory studies (Ma et al., 2020) and field‐collected samples (Ayayee et al., 2018; Pechal & Benbow, 2016; Receveur et al., 2020). All macroinvertebrate samples in this study were categorized into 10 geographic locations, seven macroinvertebrate orders, and five functional feeding groups (FFGs). Location, macroinvertebrate taxonomy, and FFG emerged as possible predictors of gut microbiome beta diversity and microbiome community composition in this study, despite there not being any significant differences in alpha diversity among the categories. FFGs (filtering collectors, gathering collectors, shredders/detritivores, scrapers, and predators) emerged as a significant predictor underscored by the differential abundances of 18 ASVs among FFGs (Figures 4b). These results suggest a clear rationale for more broadly studying how microbiomes of aquatic insects are affected by FFGs, mainly because the mechanistic basis is well developed.

Freshwater macroinvertebrate FFG categorizations are based on behavioral mechanisms of food acquisition and the type of materials consumed instead of the taxonomic designation of macroinvertebrates (Cummins, 2021; Gökçe, 2018). This approach allows for classifying hundreds of taxonomically different aquatic macroinvertebrates into relevant ecological units based on how they function and acquire food in aquatic ecosystems. Additionally, significant physiological differences exist among the various macroinvertebrate FFGs, further making them critical physiological units that can be relevant for structuring gut microbiomes of aquatic insects; a rationale proposed almost two decades ago (Harris, 1993). Thus, the different gut conditions in the FFGs in this study may be driving resulting differences in associated gut microbiomes among freshwater macroinvertebrates. This is supported by the assertion that filter feeders (Trichoptera and Diptera) tend to have slightly acidic to alkaline gut pHs (Anderson & Cargill, 1987; Cummins, 1979; Martin et al., 1980; Martin, Martin, et al., 1981), grazers/collectors that feed on biofilm, such as Baetidae and Leptophlebiidae (order Ephemeroptera) tend to have neutral to slightly alkaline gut pHs (Austin & Baker, 1988), and predatory freshwater macroinvertebrates tend to have very alkaline gut pHs than other FFGs (Anderson & Cargill, 1987; Tierno de Figueroa et al., 2011).

The FFG classification is not without its drawbacks. As with any categorical classification, there can be substantial variation among individuals within a category. Most studies with aquatic insects can confidently identify taxa down to the order or family level. However, at these levels, there may be multiple FFGs within taxa, in addition to ontogenetic variation, and this can further complicate the assessment of gut microbiota. Overall, it stands to reason that FFGs with their inherent differences in gut physiology and digestive requirements (despite some overlap in food consumed) can serve as a good delineator of freshwater macroinvertebrate gut microbiomes.

While the possible mechanistic basis for microbiome variation among FFGs is well developed and supported by our data, as well as other studies (Ayayee et al., 2018; Receveur et al., 2020), there is also substantial variation among taxa and sites, but the mechanistic basis for this variation is less understood. Given the gradient of environmental conditions that our sites represent (e.g., ranging 11 degrees of latitude and 16 °C in mean annual temperature), it seems likely that there are also considerable variations in the source pool of microbiota among sites. One fascinating result is the presence of four bacterial ASVs in high abundances in LEWI and not in the other nine locations. Lewis Run (LEWI) is an aquatic NEON field site located about 60 miles west of Washington, D.C., in Clarke County, Virginia. The site is a small wadeable stream draining a watershed of 11.9 km^2^ (2940 acres). Most of the stream reach flows through and through land managed by Casey Trees, a non‐profit organization that raises trees for planting in and around the Washington, D.C. area. The surrounding region comprised general land use types, including successional fields, pastures, woodlands, and small ponds. This site is located within NEON's Mid‐Atlantic Domain (D02), a densely populated region bounded by the Atlantic Ocean on the east and stretching down the Eastern Seaboard from southern New Jersey to northern Georgia (https://www.neonscience.org/field‐sites/lewi). It remains unclear how impacted, or unimpacted this site is relative to the other nine sites. Several studies have previously reported differences in environmental microbiome diversity and composition among different streams varying in quality (Atashgahi et al., 2015; Medeiros et al., 2016) and among sites along streams (Drury et al., 2013; Kroetsch et al., 2020; Wakelin et al., 2008), as well as reported differences between bacterioplankton and sediment partitions within streams (Ayayee et al., 2018; Fang et al., 2017; Hosen et al., 2017).

The detection of *Wolbachia* in five of the freshwater macroinvertebrate orders in this study (Coleoptera, Odonata, Trichoptera, Plecoptera, and Ephemeroptera) supports previous work that concluded that *Wolbachia* could be considered a common endosymbiont of aquatic insects, with an incidence rate of 52% (Sazama et al., 2017). The detection of *Wolbachia* in Ephemeroptera, in this study, as in other studies (Sazama et al., 2017), was low compared to the other orders it was found in (low abundance from the microbiome data and no detection from the *wsp*‐PCR study), whereas Plecoptera and Trichoptera were well represented in both approaches. However, in contrast to previous studies, we did not detect *Wolbachia* from Diptera via PCR or Diptera from the microbiome dataset. The prominent Dipteran families in this study were *Chironomidae*, *Dixidae*, *Psychodidae*, *Simuliidae*, and *Tipulidae*. We did not have any members of the Dipteran *Culicidae* family (known to harbor several pathogens of public health importance) in our dataset. There is a need to examine further the presence of *Wolbachia* across freshwater macroinvertebrate taxa and aquatic Dipteran taxa that are of ecological importance and not merely medical importance.

Finally, the interplay between deterministic processes (non‐random, species trait, niche‐based mechanistic processes) and stochastic events (ecological processes, geographic location) in shaping microbiomes (host‐associated and free‐living) (Jizhong & Daliang, 2017) are increasingly being evaluated and considered in discussions of factors shaping the gut microbiome of insects. These insights have generated various conceptual frameworks to assess dynamics governing community assembly (Vellend, 2010) and by extension, gut microbial community assembly in insect hosts (Brown et al., 2020). Of particular interest is the co‐evolution between insect hosts (terrestrial or aquatic) and associated gut microbiomes and more importantly, the significance of the ecological designation (Functional feeding groups, FFGs) versus taxonomic designation of insect hosts on shaping gut microbiomes. The results from our study perhaps lend support to deterministic mechanisms for gut microbiome assembly in freshwater macroinvertebrates, structured by ecological categorizations (functional feeding groups).

In conclusion, our study provides data in support of the existence of intrinsic processes that screen microbes from the surrounding source microbiota pool in streams before colonization and establishment in freshwater macroinvertebrate guts, predominantly underscored by the ecological classification of macroinvertebrates based on the mode of feeding, i.e., FFGs in these streams. As already mentioned, these processes may differ among FFGs due to the differences in gut physiologies, resulting in different gut microbiomes. Next, we determined that the sampled streams also contributed to the observed variations in the gut microbiome composition of the sampled macroinvertebrates. Differences in site characteristics (geographic location of stream, stream condition, pH, salinity, etc.) may be impacting aquatic macroinvertebrate gut microbiomes, mainly via influencing the source microbiota pool in the water column (bacterioplankton) or the biofilm (on leaves, twigs, rocks, debris, etc.), in these streams. Finally, we determined that the taxonomy (order, family, and genus) of the sampled macroinvertebrates in this study accounted for the lowest variation in observed microbiome community composition. Overall, results suggest that future studies characterizing freshwater macroinvertebrate gut microbiomes would be best served by focusing on sampling representatives of multiple functional feeding groups within the same streams and from multiple streams, if possible.

## AUTHOR CONTRIBUTIONS

**Paul Akwettey Ayayee:** Conceptualization (equal); data curation (equal); formal analysis (equal); funding acquisition (equal); investigation (equal); methodology (equal); project administration (equal); resources (equal); supervision (equal); validation (equal); writing – original draft (equal); writing – review and editing (equal). **Jeff Wesner:** Conceptualization (equal); data curation (equal); formal analysis (equal); funding acquisition (equal); investigation (equal); methodology (equal); project administration (equal); software (equal); supervision (equal); validation (equal); writing – original draft (equal); writing – review and editing (equal). **Scot Ouellette:** Funding acquisition (equal); investigation (equal); supervision (equal); writing – original draft (equal); writing – review and editing (equal).

## Supporting information


Appendix S1.

## Data Availability

Data and code are available at https://github.com/jswesner/neon_microbiome and will be permanently archived with a DOI via Zenodo following acceptance.
