# Supplementary material for: Geography, taxonomy, and ecological guild: Factors impacting freshwater macroinvertebrate gut microbiomes
Source: Ecol Evol. 2022 Dec 23;12(12):e9663. doi: 10.1002/ece3.9663 (PMC9789321; doi:10.1002/ece3.9663)
Supplement: Supplementary file 1 — Appendix S1. [file ECE3-12-e9663-s001.docx]

**Supplementary Information:** Geography, taxonomy, and ecological guild: factors impacting freshwater macroinvertebrate gut microbiomes.

Paul A. Ayayee^1^, Jeff S. Wesner^2^, Scot P. Ouellette^3^

^1^ Department of Biology, University of Nebraska at Omaha, Omaha, NE, USA.

^2^ Department of Biology, University of South Dakota, Vermillion, SD, USA

^3^ Department of Pathology and Microbiology, College of Medicine, University of Nebraska Medical Center, Omaha, NE, USA.

Corresponding author: Paul Ayayee. Email: [payayee@unomaha.edu](mailto:payayee@unomaha.edu)

**Prior Selection**

For modeling beta diversity, we chose priors that generated relatively flat probabilities for each possible value of beta diversity (0 to 1). Those are shown in Figure S1 along with the posterior probabilities.


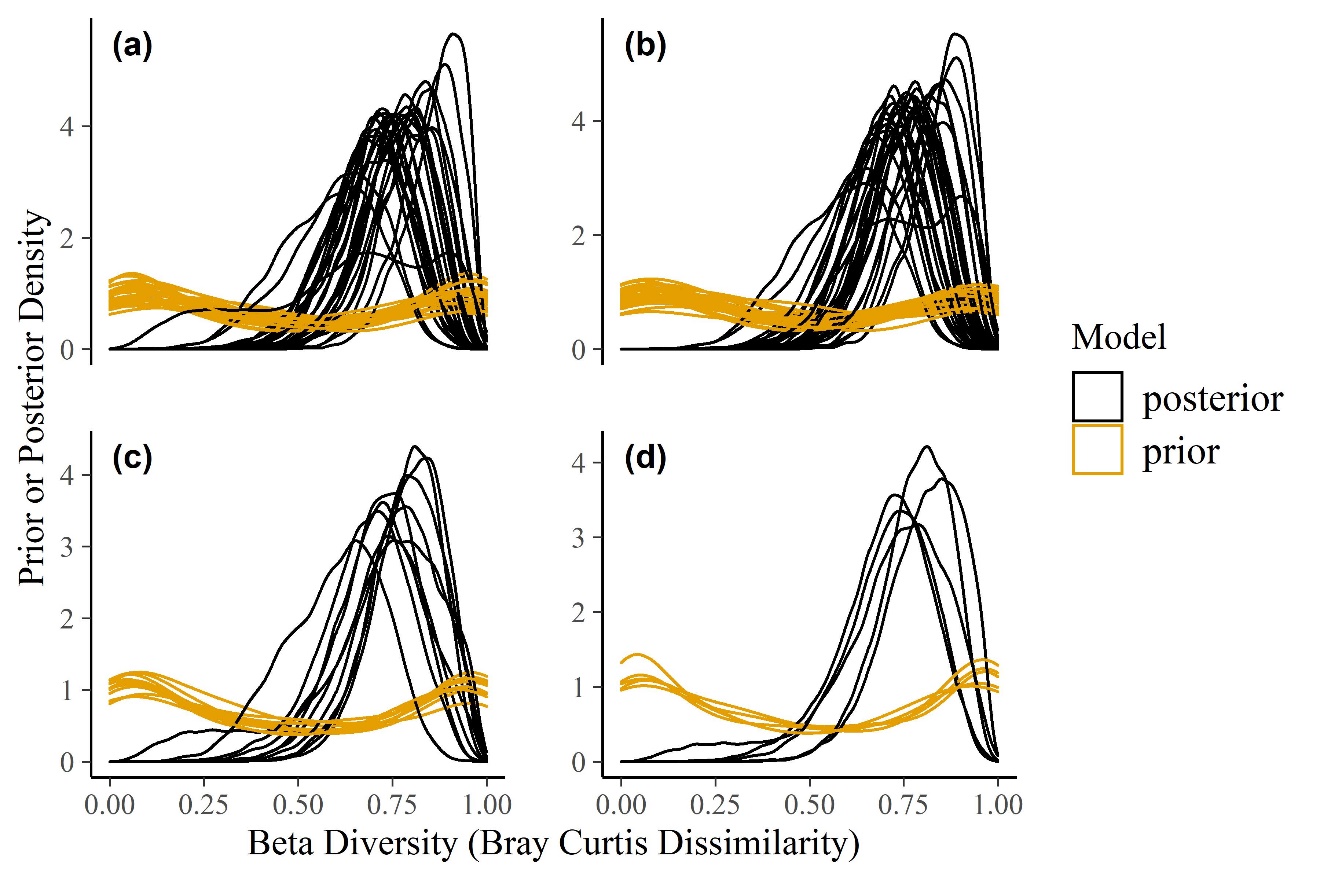


Figure S1. Comparison of prior and posterior distributions of beta diversity for a) family, b) genus, c) stream location, and d) functional feeding group. Each line represents the prior or posterior prediction or a given level of the group (e.g., each family or genera, etc).

**Model Fit**

We checked model fit by comparing the posterior predictive distribution to the distribution of the raw data. These are visual comparisons (Gabry et al. 2018) in which good model fits can simulate data that resemble the raw data. This is demonstrated by Figure S2, in which ten simulated datasets follow distributions that cluster around the raw dataset.


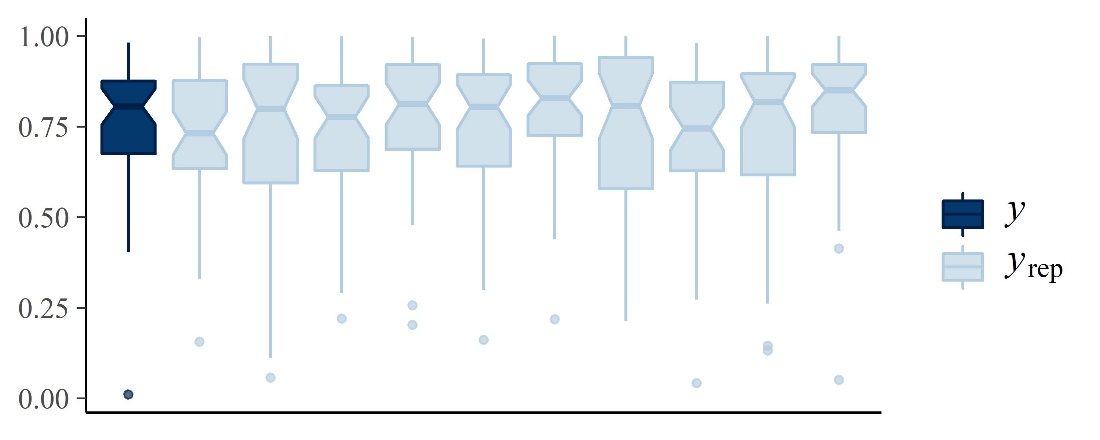


Figure S2. The ten datasets simulated from the posterior (*y*_rep_) are similar to the raw dataset (*y*). This indicates good model fit since the model is able to reproduce data that are similar to the original dataset.
